# Hidden Malnutrition in the GLP-1 Era: Micronutrient Status, Protein Adequacy, and Lean Mass as Emerging Nutritional Considerations—A Narrative Review

**DOI:** 10.3390/nu18172757

**Published:** 2026-08-23

**Authors:** Tamara Sorić, Ana Sarić, Andrija Ivanišin, Mario Lovrić, Mirta Milić, Martina Matovinović, Marijana Matek Sarić

**Affiliations:** 1 Psychiatric Hospital Ugljan, Otočkih dragovoljaca 42, 23275 Ugljan, Croatia; 2 School of Medicine, Catholic University of Croatia, Ilica 242, 10000 Zagreb, Croatia; asaric1@unicath.hr; 3 Independent Researcher, 10000 Zagreb, Croatia; andrija.ivanisin@gmail.com; 4 Institute for Anthropological Research, Ljudevita Gaja 32, 10000 Zagreb, Croatia; mario.lovric@inantro.hr; 5 Faculty of Food Technology Osijek, Josip Juraj Strossmayer University of Osijek, Franje Kuhača 18, 31000 Osijek, Croatia; 6 Lisbon Council, 1040 Brussels, Belgium; 7 Division of Toxicology, Institute for Medical Research and Occupational Health, Ksaverska cesta 2, 10001 Zagreb, Croatia; mmilic@imi.hr; 8 Department of Internal Medicine, Division of Metabolic Diseases, University Hospital Centre Zagreb, Kišpatićeva 12, 10000 Zagreb, Croatia; mmatovin@kbc-zagreb.hr; 9 Faculty of Kinesiology, University of Zagreb, Horvaćanski zavoj 15, 10000 Zagreb, Croatia; 10 Department of Health Studies, University of Zadar, Splitska 1, 23000 Zadar, Croatia; marsaric@unizd.hr

**Keywords:** GLP-1 receptor agonists, obesity, nutritional assessment, body composition, sarcopenic obesity, protein intake, micronutrients, hidden malnutrition, nutritional monitoring, patient-centered care

## Abstract

Glucagon-like peptide-1 (GLP-1) receptor agonists and dual glucose-dependent insulinotropic polypeptide (GIP)/GLP-1 receptor agonists have transformed obesity management by producing substantial, sustained weight loss and improving metabolic health. Alongside these benefits, their effects on appetite, food intake, and gastrointestinal function have raised increasing interest in the nutritional consequences of pharmacologically induced weight loss. Although reductions in energy intake contribute to therapeutic efficacy, they may also influence protein intake, dietary quality, micronutrient adequacy, and skeletal muscle health, particularly in individuals with pre-existing nutritional vulnerability. This narrative review summarizes current evidence regarding nutritional considerations during GLP-1-based therapy, including changes in dietary intake, protein and micronutrient adequacy, body composition, muscle health, and nutritional assessment. Particular attention is given to populations at increased nutritional risk, practical approaches to nutritional monitoring, and strategies to support adequate nutrition during treatment. Current evidence suggests that nutritional responses to GLP-1-based therapy are heterogeneous and cannot be adequately evaluated using body weight alone. Assessment of dietary intake, body composition, muscle function, physical performance, and individual clinical characteristics provides a more comprehensive understanding of nutritional status than anthropometric measures alone. While routine supplementation or intensive monitoring is not supported for all patients, a risk-based and individualized approach appears appropriate, particularly for older adults and individuals with sarcopenic obesity, previous bariatric surgery, chronic gastrointestinal disease, or persistent treatment-related gastrointestinal symptoms. Future research should establish clinically meaningful nutritional outcomes and determine which nutritional interventions improve patient-centered outcomes during long-term obesity management.

## 1. Introduction

The introduction of glucagon-like peptide-1 (GLP-1) receptor agonists and dual glucose-dependent insulinotropic polypeptide (GIP)/GLP-1 receptor agonists has fundamentally changed the treatment of obesity and type 2 diabetes (T2D). Clinical trials of semaglutide and tirzepatide have demonstrated weight reductions approaching 15–20% of initial body weight, accompanied by improvements in glycemic control, blood pressure, lipid profiles, and cardiovascular risk markers [1,2,3,4]. These outcomes have positioned GLP-1-based therapies among the most effective non-surgical interventions available for obesity management.

The clinical success of these agents has shifted attention beyond weight loss efficacy alone toward the broader health consequences of pharmacologically induced weight reduction. While reductions in adipose tissue are associated with substantial metabolic benefits, accumulating evidence suggests that weight loss achieved during GLP-1 therapy is not limited to fat mass. Body composition analyses from clinical trials and observational studies indicate that a proportion of total weight loss may be attributable to fat-free mass [5,6,7]. Although some reduction in lean tissue is expected during weight loss, the clinical relevance of these changes depends on the extent to which they involve skeletal muscle and affect muscle function, particularly among older adults and individuals with pre-existing sarcopenia, frailty, or chronic disease [6,8,9].

At the same time, GLP-1 receptor agonists exert their therapeutic effects largely through appetite suppression, delayed gastric emptying, and enhanced satiety. Many patients report reduced hunger, early meal termination, nausea, food aversions, and other gastrointestinal symptoms that contribute to lower energy intake [10,11]. While these effects support weight reduction, they may also influence dietary quality and nutritional adequacy. Nutritional outcomes were not primary endpoints in most pivotal GLP-1 trials, and relatively little attention has been directed toward the potential consequences of sustained reductions in food intake on protein consumption, micronutrient status, and overall nutritional health.

These concerns are particularly relevant given that obesity itself is frequently accompanied by nutritional inadequacies despite excessive energy intake. Deficiencies in vitamin D, iron, folate, vitamin B12, magnesium, zinc, and other micronutrients have been consistently reported in individuals with obesity [12,13,14]. The concept of “overfed but undernourished” reflects the observation that excess adiposity does not necessarily indicate adequate nutritional status. Consequently, individuals initiating GLP-1 therapy may already have pre-existing nutritional vulnerabilities that could be exacerbated by further reductions in food intake during treatment.

Adequate dietary protein intake is another important, though comparatively underinvestigated, aspect of nutritional care during GLP-1 therapy. It is essential for maintaining muscle protein synthesis, preserving lean body mass, and supporting physical function during weight loss. The appetite suppression, early satiety, reduced energy intake, gastrointestinal adverse effects, and changes in food preferences commonly observed during GLP-1 therapy may compromise protein intake in some individuals, particularly when nutritional counseling is not provided [11,15,16]. When combined with insufficient resistance exercise, inadequate protein consumption may contribute to accelerated losses of skeletal muscle and the development of sarcopenic obesity, a condition characterized by the coexistence of excess adiposity and impaired muscle quantity or function [17,18]. This phenotype has been associated with poorer functional outcomes, increased frailty, reduced quality of life, and greater healthcare utilization [17,19].

Despite increasing discussion of these issues in clinical practice, direct prospective evidence establishing clinically important micronutrient deficiencies, protein inadequacy, or functional muscle loss as consequences of GLP-1-based therapy remains limited. These outcomes are therefore considered throughout this review as potential nutritional vulnerabilities or emerging concerns rather than established consequences of treatment. Most published studies have focused on weight loss, glycemic control, and cardiometabolic outcomes, whereas nutritional adequacy, micronutrient status, body composition changes, and long-term functional consequences have received considerably less attention. Furthermore, no consensus currently exists regarding nutritional monitoring strategies, optimal protein targets, laboratory surveillance, or the role of targeted supplementation during GLP-1 therapy.

As the use of GLP-1 receptor agonists and dual GIP/GLP-1 receptor agonists continues to expand globally, understanding their nutritional implications is becoming increasingly important. Successful obesity treatment should be evaluated not only by the magnitude of weight loss achieved but also by the preservation of nutritional status, skeletal muscle health, and long-term metabolic resilience. This narrative review examines the emerging concept of hidden malnutrition in the era of GLP-1 therapy, integrating current evidence on micronutrient status, protein adequacy, changes in lean body mass, and sarcopenic risk within a unified clinical and public health framework. It also discusses high-risk populations, public health implications, and practical approaches to individualized nutritional assessment, monitoring, and intervention during treatment.

For the purposes of this review, “hidden malnutrition” is used as an integrating clinical concept to describe nutritional vulnerability that may not be apparent from body weight or body mass index alone, rather than as a single clinical outcome or diagnostic entity. Within this framework, dietary inadequacy refers to habitual intake of energy, protein, or specific micronutrients that is insufficient relative to estimated nutritional requirements, whereas nutrient deficiency refers to an established deficiency of a specific nutrient based on appropriate biochemical and/or clinical indicators [20]. Disease- or treatment-related malnutrition refers to malnutrition arising in the context of reduced intake or assimilation, inflammation, or other disease- or treatment-associated factors, in accordance with established diagnostic frameworks [21]. Lean-mass loss, skeletal muscle loss, sarcopenia, and frailty represent related but distinct constructs: changes in lean mass should not be equated with loss of skeletal muscle; sarcopenia is characterized primarily by reduced muscle strength, with low muscle quantity or quality supporting the diagnosis [22]; and frailty describes a broader state of reduced physiological reserve and increased vulnerability to stressors [23]. Gastrointestinal symptom-related dietary restriction is considered a potential contributor to nutritional vulnerability rather than a form of malnutrition itself [21]. Throughout this review, these terms are therefore used according to their specific clinical meaning and are not treated as interchangeable manifestations of “hidden malnutrition.”

## 2. Literature Search and Evidence Synthesis

This narrative review was designed to provide a broad, clinically oriented synthesis of the literature on nutritional considerations during GLP-1-based therapy. The literature search was undertaken to identify relevant evidence across the principal thematic areas of the review and was not conducted as a systematic or scoping review.

The evidence presented in this review was identified through searches of PubMed/MEDLINE, Scopus, and Web of Science. The primary literature search, covering publications from database inception, was completed on 31 March 2026, with emphasis on recent evidence reflecting the rapidly evolving landscape of GLP-1-based therapies for obesity and T2D. Earlier publications were included as needed to contextualize concepts related to obesity-associated malnutrition, sarcopenia, protein adequacy, and changes in body composition during weight loss. Targeted supplementary searches were conducted during manuscript revision on 13 August 2026 to identify newly available evidence and additional literature relevant to specific topics addressed in the review.

The literature search was conducted using a combination of keywords and Medical Subject Headings (MeSH) terms related to GLP-1 therapies, nutritional status, and body composition. Search terms included: “GLP-1 receptor agonist”, “GLP-1RA”, “semaglutide”, “tirzepatide”, “liraglutide”, “dual incretin agonist”, “obesity pharmacotherapy”, “hidden malnutrition”, “malnutrition”, “micronutrient deficiency”, “vitamin D”, “vitamin B12”, “iron deficiency”, “magnesium”, “calcium”, “protein intake”, “protein inadequacy”, “lean body mass”, “skeletal muscle”, “muscle loss”, “sarcopenia”, “sarcopenic obesity”, “frailty”, “body composition”, “diet quality”, “nutritional assessment”, “resistance training”, and “clinical nutrition”. Search terms were combined using Boolean operators (AND, OR), and the reference lists of key publications were examined to identify additional relevant studies.

A broad range of evidence sources was considered, including randomized controlled trials, observational studies, systematic reviews, meta-analyses, clinical practice guidelines, consensus statements, and other peer-reviewed publications available in English. Preference was given to studies directly evaluating nutritional status, dietary intake, body composition, muscle preservation, and micronutrient adequacy among individuals receiving GLP-1-based therapies. Publications lacking sufficient methodological information or unavailable as full-text articles were not considered. Literature discussed in this review was selected based on its relevance to the thematic scope of the review, recency, and study quality rather than according to a pre-specified systematic screening protocol. Literature identification and selection were undertaken by three authors (T.S., A.S., and M.M.S.).

Because the objective of this review was to provide an integrated overview of the topic, findings were synthesized narratively rather than quantitatively. The strength and relevance of the evidence were considered according to study design, sample size, methodological quality, directness to the population and outcomes addressed in this review, and consistency of findings across studies. Particular attention was given to potential sources of bias and confounding when interpreting observational evidence. Evidence from randomized trials, systematic reviews and meta-analyses, clinical guidelines, and larger prospective studies was generally given greater weight, while smaller observational, cross-sectional, and mechanistic studies were interpreted more cautiously and primarily as supportive or hypothesis-generating evidence. Where findings were inconsistent, interpretation considered differences in study populations, treatment duration, outcome assessment, sample size, control for confounding, and methodological limitations. These considerations informed the narrative interpretation of the evidence rather than constituting a formal study-level quality rating or certainty-of-evidence assessment.

The identified literature was organized into thematic domains that reflect the principal aims of the review, including mechanisms underlying nutritional vulnerability during GLP-1 therapy; micronutrient deficiencies; inadequate protein intake; loss of lean body mass; sarcopenic obesity; diet quality; individualized nutritional management; supplementation strategies; and public health implications.

**Figure 1:**
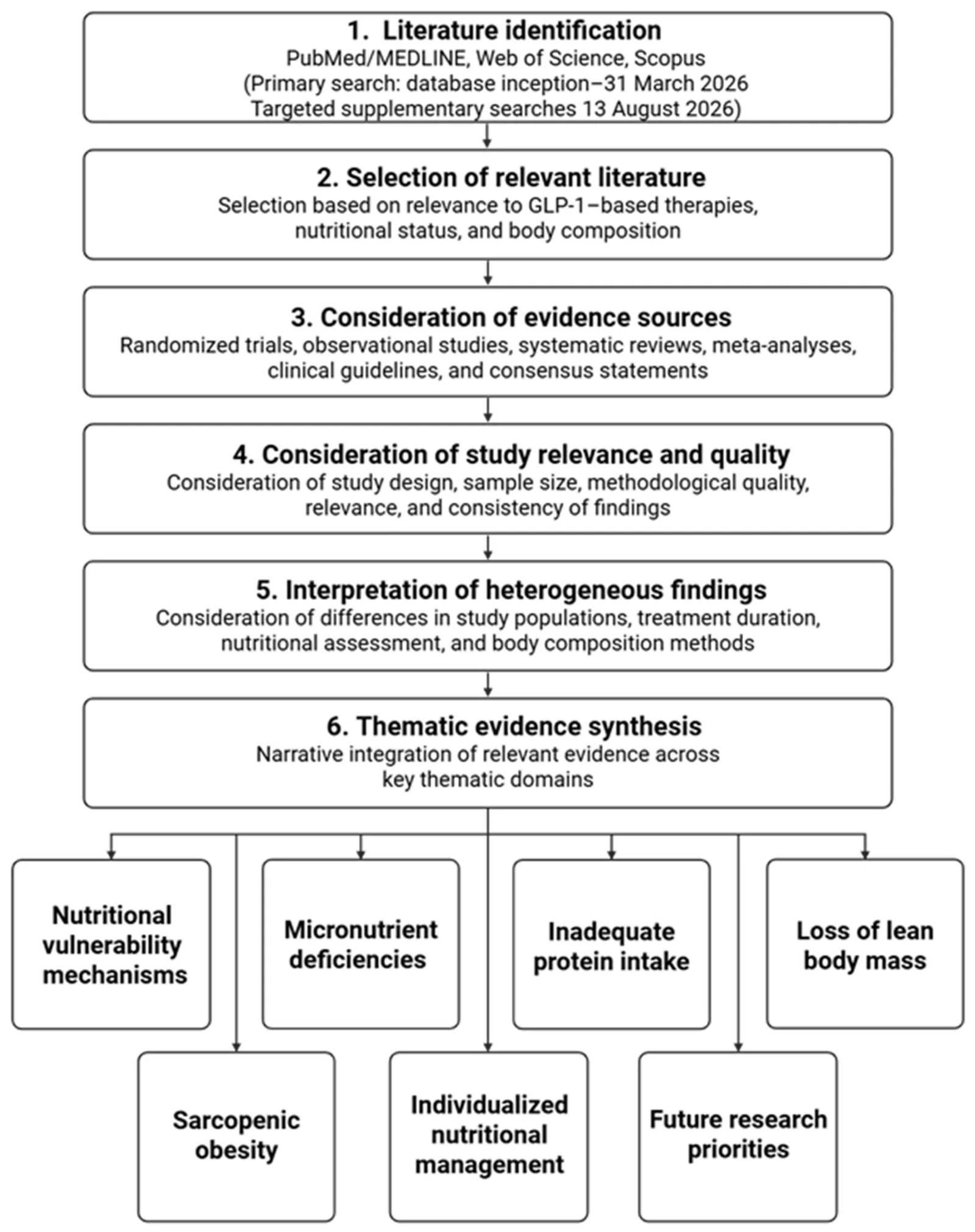
A conceptual overview of the literature search and evidence synthesis process used in this narrative review. The figure illustrates the approach to literature identification, selection, and thematic synthesis and is not intended to represent a PRISMA-type systematic study-selection process. Abbreviations: GLP-1, glucagon-like peptide-1. Created in BioRender. Milić, M. (2026), https://BioRender.com/uwsi2cc (accessed on 13 August 2026).

A conceptual overview of the literature search and thematic evidence synthesis process is presented in Figure 1.

## 3. Mechanisms Underlying Nutritional Vulnerability During GLP-1 Therapy

The mechanisms underlying the therapeutic efficacy of GLP-1-based therapies may also contribute to nutritional vulnerability. This concept extends beyond reductions in energy intake alone and encompasses a broader set of physiological and behavioral changes that may influence dietary adequacy, protein consumption, body composition, and micronutrient status. Nutritional risk during GLP-1 therapy is unlikely to be determined solely by pharmacological treatment itself, but rather by the interaction between medication-induced changes in eating behavior and an individual’s baseline nutritional status, age, comorbidities, dietary habits, physical activity patterns, functional reserve, and other lifestyle-related factors [13,24,25]. Sleep duration and circadian health may also influence appetite regulation, energy balance, glucose homeostasis, and overall metabolic health [26,27], although their specific interaction with GLP-1-based therapies and potential impact on nutritional outcomes remain insufficiently investigated.

GLP-1 receptor agonists promote weight loss primarily through appetite suppression, enhanced satiety, and reductions in food reward [28,29]. Activation of central pathways involved in appetite regulation decreases hunger and reduces motivation to eat, leading to substantial reductions in daily energy intake [29,30]. Delayed gastric emptying further contributes to early satiety and smaller meal sizes, particularly during the initial phases of treatment [31]. These mechanisms are important for achieving clinically meaningful weight loss. From a nutritional perspective, sustained reductions in food intake may increase the likelihood of inadequate consumption of protein and essential micronutrients if dietary choices are not carefully planned [11,16].

Gastrointestinal adverse effects may further influence dietary behavior. Nausea, vomiting, abdominal discomfort, bloating, and early satiety are among the most frequently reported side effects of GLP-1 therapy and represent common reasons for treatment discontinuation [2,32]. Even when symptoms are mild, they may alter food selection and eating patterns. Patients often report avoiding foods perceived as difficult to tolerate, consuming smaller portions, skipping meals, or relying on a limited range of foods during periods of gastrointestinal discomfort. Such adaptations may reduce dietary variety and unintentionally lower nutrient intake [16,24,33].

The effect of GLP-1 therapy on food preferences and dietary quality is therefore a growing area of interest. Beyond reducing overall food intake, GLP-1 signaling appears to influence food-related reward pathways and eating behavior [28,34,35]. Several studies have reported reduced preference for highly processed, energy-dense foods, decreased food cravings, and alterations in ingestive behavior during treatment [34,36]. Whether these changes ultimately improve overall dietary quality remains unclear. Evidence describing shifts in dietary patterns, food-group consumption, and nutrient intake during GLP-1 therapy remains surprisingly limited, despite the widespread use of these medications [16,24,33]. It is also uncertain whether changes in food preferences inadvertently reduce consumption of protein-rich foods or other important sources of micronutrients [16,24]. Current evidence does not establish that GLP-1-induced changes in food reward selectively reduce the consumption of protein-dense or micronutrient-rich foods rather than reflecting broader changes in appetite, food preference, and total energy intake [37]. Whether selective nutrient avoidance occurs during treatment therefore remains an important knowledge gap requiring further investigation.

The nutritional implications of reduced food intake are particularly relevant because many individuals initiating GLP-1 therapy may already have underlying nutritional vulnerabilities. Excess adiposity does not necessarily indicate nutritional adequacy, and obesity frequently coexists with suboptimal diet quality and other factors that increase nutritional risk [12,13]. Consequently, medication-induced reductions in food intake may exacerbate pre-existing nutritional challenges in susceptible individuals. Recent reviews have highlighted that sustained reductions in dietary intake during GLP-1 therapy could further compromise nutritional adequacy if not accompanied by appropriate dietary guidance and nutritional support, although robust long-term data regarding dietary quality and micronutrient adequacy remain scarce [11,16].

Protein inadequacy has emerged as one of the principal nutritional concerns associated with GLP-1 therapy. Although direct evidence evaluating protein intake among GLP-1 users remains limited, several expert consensus statements and nutrition-focused reviews have highlighted the potential for inadequate protein consumption, particularly among individuals experiencing marked appetite suppression, gastrointestinal symptoms, or rapid weight loss [11,24,25]. Most clinical studies of GLP-1-based therapies have focused primarily on weight loss, glycemic control, and cardiometabolic outcomes, whereas dietary composition, nutrient intake, and broader measures of nutritional adequacy have received considerably less attention [16,33]. Current concerns are therefore driven largely by biological plausibility, observational data, and established knowledge regarding protein requirements during weight loss. The biological rationale for protein inadequacy during GLP-1-based therapy is strong, whereas direct clinical evidence establishing its prevalence and consequences remains limited.

Recent dietary intake data provide additional support for these concerns. In an observational study of adults receiving GLP-1 receptor agonists or GIP/GLP-1 receptor agonists, average reported energy intake was substantially below estimated requirements, while protein and fiber intakes frequently failed to meet recommended targets [38]. Although these findings require confirmation in larger prospective cohorts, they suggest that reductions in food intake during therapy may not always be accompanied by sufficient attention to nutrient density and dietary quality. Given the observational nature of these data, however, inadequate reported intake should not be interpreted as evidence of clinically established malnutrition.

Physical activity may substantially modify nutritional and body composition outcomes during GLP-1 therapy. Resistance exercise is widely recognized as one of the most effective strategies for preserving skeletal muscle during weight loss and is increasingly recommended alongside adequate protein intake in individuals receiving GLP-1-based treatments [24,39]. Despite this, relatively little is known about exercise behaviors among GLP-1 users or the extent to which physical activity may mitigate losses of lean tissue during treatment. The contribution of physical activity to body composition outcomes during GLP-1 therapy remains poorly characterized and warrants further investigation.

Hidden malnutrition should not be viewed as an inevitable consequence of GLP-1 therapy. Rather, it provides a framework for considering potential nutritional vulnerabilities that may emerge in susceptible individuals, particularly when reduced food intake occurs in the context of pre-existing nutritional risk. Certain populations may be particularly vulnerable during periods of negative energy balance, including older adults, individuals with pre-existing sarcopenia or frailty, patients with chronic disease, and those with low habitual protein intake [6,11,15,17]. Aging-related anabolic resistance further increases dietary protein requirements to maintain skeletal muscle health, potentially amplifying the effects of reduced intake [15,40,41]. Baseline nutritional status may therefore play an important role in determining individual susceptibility to adverse nutritional outcomes during GLP-1 therapy.

The magnitude and pace of weight loss may also influence nutritional risk. Individuals experiencing rapid weight loss may be particularly susceptible to inadequate nutrient intake and unfavorable changes in body composition if nutritional support is not provided. Although some reduction in lean mass is expected during successful weight loss, insufficient protein intake combined with physical inactivity may increase the risk of adverse changes in body composition and skeletal muscle health, particularly among vulnerable populations [15,40,41].

Reduced protein intake, loss of lean body mass, and physical inactivity may collectively contribute to the development or progression of sarcopenic obesity, a condition characterized by the coexistence of excess adiposity and impaired muscle quantity or function [17,42,43]. Given the established associations between sarcopenic obesity, frailty, functional decline, and adverse health outcomes, preserving skeletal muscle should be considered an important component of successful obesity treatment rather than a secondary consideration [17,44,45].

Current evidence does not suggest that GLP-1 receptor agonists induce clinically significant malabsorption comparable to that observed following bariatric surgery. Nutritional concerns associated with GLP-1-based therapies appear to arise predominantly from reduced food intake, altered eating behaviors, and suboptimal dietary quality rather than impaired nutrient absorption [46,47]. Although awareness of these nutritional considerations is increasing, many aspects of nutritional health during GLP-1 therapy remain incompletely characterized. In particular, prospective data on dietary quality, micronutrient status, protein adequacy, body composition trajectories, and long-term functional outcomes remain limited [11,16,24].

**Figure 2:**
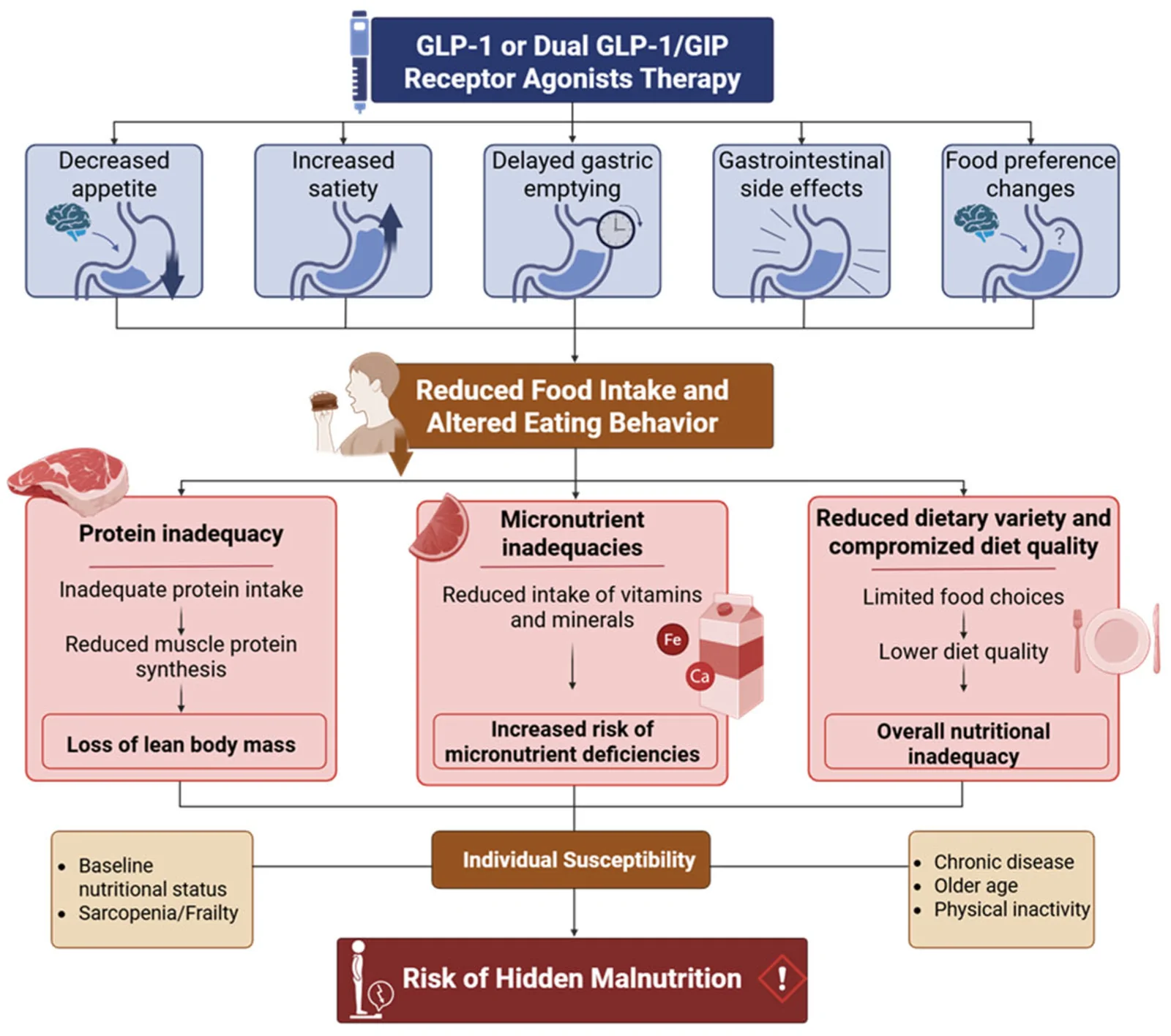
Mechanisms underlying nutritional vulnerability during GLP-1 therapy. Abbreviations: GLP-1, glucagon-like peptide-1; GLP-1/GIP, glucagon-like peptide-1/glucose-dependent insulinotropic polypeptide. Created in BioRender. Milić, M. (2026), https://BioRender.com/5lbty3n (accessed on 13 August 2026).

The rapid clinical adoption of GLP-1-based therapies has therefore outpaced research examining their nutritional implications. Hidden malnutrition offers a clinically relevant framework for interpreting nutritional risk during pharmacologically induced weight loss, emphasizing that treatment success cannot be judged solely by the magnitude of weight reduction. Instead, dietary quality, protein adequacy, micronutrient status, body composition, and physical function should be considered complementary components of comprehensive obesity care throughout treatment (Figure 2).

## 4. Micronutrient Status During GLP-1 Therapy

Micronutrient status is an increasingly important consideration in the nutritional management of individuals receiving GLP-1-based therapy. Although the efficacy of these agents in promoting weight loss and improving cardiometabolic health has been extensively investigated, considerably less is known about their effects on micronutrient intake and nutritional adequacy. Sustained reductions in energy intake may compromise the intake of essential vitamins and minerals if dietary quality is not maintained, particularly during prolonged treatment or rapid weight loss [11,16].

Evidence describing micronutrient status during GLP-1 therapy remains limited [11,16]. Nutritional outcomes were not predefined endpoints in most pivotal randomized clinical trials evaluating semaglutide, liraglutide, or tirzepatide, and systematic biochemical assessment of micronutrient status has rarely been incorporated into study protocols [1,2,4,48,49,50,51]. As a result, current knowledge largely relies on observational studies, retrospective analyses, dietary intake assessments, and expert consensus documents rather than on prospective trials specifically designed to evaluate nutritional outcomes [11,16,24]. In a large retrospective claims-based cohort, new nutritional deficiency diagnoses were recorded in approximately 22% of GLP-1RA users within 12 months of treatment initiation, with vitamin D deficiency being the most frequently documented abnormality, followed by B-vitamin deficiencies and anemia [52]. Patients with previously coded nutritional deficiencies were excluded; however, systematic biochemical measurements before and after treatment were not available. These findings should therefore be interpreted as incident coded diagnoses rather than confirmed treatment-emergent biochemical deficiencies.

Interpretation of nutritional abnormalities during GLP-1-based therapy requires distinction between pre-existing nutritional problems and treatment-emergent changes. Individuals with obesity and T2D may already have suboptimal dietary quality or micronutrient deficiencies before treatment initiation [12,13], making attribution to pharmacotherapy difficult when pretreatment nutritional status is unavailable. Vitamin D deficiency is particularly relevant in this context, as it is more common among individuals with obesity than among those with normal body weight. A systematic review and meta-analysis by Pereira-Santos et al. [53] reported a 35% higher prevalence of vitamin D deficiency in individuals with obesity. Therefore, vitamin D deficiency documented during GLP-1-based therapy cannot be assumed to be treatment-emergent in the absence of pretreatment biochemical assessment or an appropriate comparator. More broadly, evidence should be interpreted according to whether studies report changes from baseline, incident diagnoses or comparator-based differences, or only cross-sectional findings during treatment, as these designs provide different levels of information regarding treatment-emergent abnormalities.

The nutritional implications of GLP-1 therapy appear to vary considerably among individuals. Rather than representing a predictable pharmacological effect, nutritional outcomes are likely influenced by the interaction between treatment-related reductions in food intake and patient-specific factors, including baseline nutritional status, age, dietary habits, treatment duration, gastrointestinal tolerance, concomitant medications, and underlying comorbidities. Consequently, the risk of clinically relevant micronutrient inadequacies is unlikely to be uniform across all patients, supporting an individualized approach to nutritional assessment and monitoring [39,47,54].

Several micronutrients have emerged as areas of clinical interest due to their essential roles in musculoskeletal health, hematopoiesis, neurological function, immune regulation, and cellular metabolism. Among these, vitamin D, vitamin B12, iron, calcium, magnesium, folate, and zinc have received the greatest attention in the literature [46,47]. Although deficiencies of these nutrients have been reported during GLP-1-based therapy, a direct causal relationship has not been established for most micronutrients [46,55]. Interpretation of published studies is further complicated by differences in study populations, dietary assessment methods, laboratory definitions of deficiency, treatment duration, and concomitant lifestyle interventions [16]. Current findings should be interpreted as signals of potential nutritional vulnerability rather than evidence that GLP-1-based therapy directly causes micronutrient deficiency.

Routine laboratory screening and universal micronutrient supplementation cannot currently be recommended for all individuals receiving GLP-1 receptor agonists or dual GIP/GLP-1 receptor agonists. Instead, nutritional assessment should be individualized according to baseline nutritional risk, dietary intake, persistent gastrointestinal symptoms, the magnitude and duration of weight loss, comorbidities, concomitant medications, and clinical features suggestive of deficiency. Whenever feasible, optimizing dietary quality should remain the primary strategy for maintaining adequate micronutrient intake, whereas laboratory evaluation and targeted supplementation should be reserved for individuals with documented deficiencies or those considered at increased nutritional risk, in accordance with current clinical recommendations [25,39,54].

The micronutrients most frequently discussed in relation to GLP-1 therapy, together with their clinical relevance, populations at increased risk, potential consequences of inadequate status, and practical considerations for nutritional assessment and monitoring, are summarized in Table 1.

## 5. Protein Intake During GLP-1 Therapy

Adequate protein intake represents a central component of nutritional care during GLP-1-based therapy because dietary protein supports adaptation to negative energy balance, skeletal muscle protein synthesis, and overall nutritional quality during weight reduction [15]. These considerations are particularly relevant during intentional weight loss, where insufficient protein intake may contribute to disproportionate reductions in lean tissue despite favorable reductions in adiposity. A systematic review and meta-analysis including 47 studies and 3218 adults with overweight or obesity undergoing weight loss demonstrated that higher protein intake significantly attenuated muscle mass loss, with protein intakes below 1.0 g/kg/day associated with greater reductions in muscle mass and intakes above 1.3 g/kg/day associated with more favorable body composition outcomes [73]. However, protein targets expressed relative to body weight require careful interpretation in individuals with obesity because the appropriate body-weight denominator may differ according to clinical context, and the underlying literature does not consistently distinguish actual, ideal, adjusted, or target body weight [74,75]. Protein requirements should therefore be individualized according to age, body composition, physical activity, rate of weight loss, renal function, and overall nutritional status [41,76,77].

Very high protein intakes should not be interpreted as routine targets during GLP-1-based therapy. Estimates suggesting that intakes around 2.0 g/kg/day may be tolerated and that approximately 3.5 g/kg/day represents an upper level are derived from healthy adults and well-adapted individuals and are not broadly applicable to patients with obesity or comorbid disease [78]. In older adults, protein requirements may be higher because of anabolic resistance, with expert guidance generally supporting approximately 1.0–1.2 g/kg/day in healthy older adults and 1.2–1.5 g/kg/day in many individuals with acute or chronic illness, while taking renal function and overall clinical status into account [40,41]. Patients with chronic kidney disease require separate assessment because appropriate protein intake depends on kidney function, nutritional status, dialysis status, and the risk of protein-energy wasting; high-protein targets used in otherwise healthy adults should therefore not be extrapolated to this population [79,80].

Recent dietary intake studies provide direct insights into protein consumption during GLP-1 therapy. In a cross-sectional study of 69 adults treated with GLP-1 receptor agonists for at least one month, protein intake accounted for an appropriate proportion of total energy intake; however, absolute protein intake relative to body weight frequently failed to meet recommended levels. Participants also exhibited several indicators of suboptimal dietary quality, including inadequate fiber (14.5 g/day), calcium (863 mg/day), magnesium (266 mg/day), and vitamin D (4 μg/day) intake, suggesting that insufficient protein intake may occur alongside broader nutritional inadequacies rather than as an isolated finding [33].

Comparable findings have been reported in a larger preliminary dietary assessment of 387 adults receiving GLP-1 receptor agonists or dual GIP/GLP-1 receptor agonists. Mean reported energy intake was only 753 kcal/day, accompanied by a mean protein intake of 33.4 g/day and fiber intake of 7.2 g/day. Fewer than 10% of participants achieved recommended protein intake targets, while inadequate intakes of several micronutrients were also common [38]. Although these findings should be interpreted cautiously, the reported energy and protein intakes were unusually low and may have been influenced by underreporting inherent to self-reported dietary assessment, characteristics of the selected study sample, and the preliminary nature of the study. These values should therefore not be assumed to represent typical dietary intake among individuals receiving GLP-1-based therapy. Nevertheless, the findings highlight the potential difficulty of maintaining adequate protein and nutrient intake during substantial appetite suppression.

These studies indicate that reductions in energy intake during GLP-1 therapy may not be accompanied by proportional increases in dietary protein density. Protein intake expressed as a percentage of total energy intake may appear adequate while absolute intake relative to body weight remains insufficient to support muscle preservation during weight loss [33]. These findings highlight the importance of evaluating protein intake using body weight-based recommendations rather than relying solely on macronutrient distribution. Nevertheless, the available evidence should be interpreted cautiously. Existing studies are predominantly observational, include relatively small study populations, and rely on self-reported dietary assessment methods. Moreover, protein intake has generally been evaluated as a secondary nutritional outcome rather than a predefined study endpoint. Consequently, the prevalence, determinants, and long-term clinical significance of inadequate protein intake during GLP-1 therapy remain incompletely characterized [16,33,47,54].

Beyond total protein intake, the quality and distribution of dietary protein may also influence nutritional outcomes during GLP-1 therapy. High-quality protein foods provide essential amino acids required to stimulate muscle protein synthesis while also serving as important dietary sources of micronutrients, including iron, zinc, vitamin B12, and calcium [81,82]. Therefore, inadequate consumption of these foods may contribute simultaneously to protein inadequacy and broader nutritional deficiencies. Recent expert consensus statements recommend prioritizing high-quality protein sources and distributing protein intake across meals to optimize muscle protein synthesis, particularly among older adults, individuals experiencing rapid weight loss, those with low habitual protein intake, and patients at increased risk of sarcopenia [25,47,54]. These recommendations are supported by established principles of clinical nutrition and healthy aging but have not yet been validated in prospective randomized studies specifically conducted in individuals receiving GLP-1-based therapies [25,39]. Optimizing protein intake, together with resistance exercise, may help support muscle health during pharmacological weight loss, although direct evidence supporting this combined approach in GLP-1-treated populations remains limited [83].

Future prospective studies should therefore evaluate protein intake alongside body composition, physical performance, and patient-centered clinical outcomes to establish evidence-based nutritional recommendations for individuals receiving GLP-1-based therapies.

## 6. Changes in Body Composition During GLP-1 Therapy

Changes in body composition have become a major focus of discussion surrounding GLP-1-based therapies, reflecting growing recognition that the composition of weight loss may be as clinically relevant as its magnitude. While reduction in excess adiposity remains the primary objective of obesity treatment, loss of lean tissue may have important implications for physical function, metabolic health, and long-term weight maintenance. As the clinical use of GLP-1 receptor agonists and dual GIP/GLP-1 receptor agonists continues to increase, greater attention has been directed toward their effects on body composition and skeletal muscle health [83,84].

Available evidence consistently indicates that a proportion of weight lost during GLP-1 therapy is attributable to reductions in lean mass. In the STEP 1 body composition substudy, 68 weeks of semaglutide treatment resulted in reductions of approximately 10.4 kg in fat mass and 6.9 kg in lean mass, with fat mass accounting for the majority of total tissue loss [85]. Despite this absolute decline, the proportion of lean tissue relative to total body weight increased because reductions in fat mass were considerably greater [85]. Similar findings have been reported with tirzepatide. In the SURMOUNT-1 DXA substudy, body weight decreased by 21.3%, with approximately 75% of weight loss attributable to reductions in fat mass and 25% to reductions in lean mass, resulting in an overall improvement in body composition [86]. Importantly, neither study was designed to determine whether these changes translated into adverse effects on muscle strength, physical performance, or other functional outcomes.

The observation that lean mass decreases during GLP-1 therapy should be interpreted within the broader context of weight-loss physiology. Loss of lean tissue occurs during virtually all forms of intentional weight reduction, including lifestyle interventions, pharmacotherapy, and bariatric surgery. Meta-analytic evidence suggests that approximately 20–30% of the total weight lost during conventional weight-loss interventions is fat-free mass, although substantial interindividual variability exists by age, sex, baseline adiposity, dietary composition, physical activity, and the magnitude of weight loss [87,88]. Viewed from this perspective, the lean mass reductions reported during GLP-1 therapy do not necessarily appear disproportionate relative to the degree of weight loss achieved.

Most body composition studies have relied on dual-energy X-ray absorptiometry (DXA), which presents interpretive challenges. Although DXA provides valuable information regarding changes in fat mass and lean mass, it does not directly quantify skeletal muscle and cannot distinguish skeletal muscle from other components of lean soft tissue, including organs, connective tissue, and body water compartments. Furthermore, DXA-derived estimates of lean mass are influenced by hydration status, which may affect the interpretation of longitudinal changes in body composition [89,90,91]. Reductions in measured lean mass should therefore not be assumed to reflect equivalent losses of functional skeletal muscle. The terms “lean mass,” “fat-free mass,” and “skeletal muscle mass” should not be used interchangeably, and DXA-derived changes in lean mass should not be equated with changes in skeletal muscle mass. Several recent reviews have emphasized that changes in lean mass may overestimate true muscle loss and that body composition data should ideally be interpreted alongside assessments of muscle strength, physical performance, and muscle quality [83,84].

This distinction is particularly relevant because muscle quantity and muscle quality are not synonymous. Obesity is frequently associated with ectopic fat accumulation within skeletal muscle (myosteatosis), a phenomenon that has been linked to impaired muscle function, reduced mobility, insulin resistance, and adverse metabolic outcomes [92,93]. Weight loss may therefore reduce intramuscular and intermuscular adipose tissue while simultaneously decreasing overall muscle volume. Recent magnetic resonance imaging analyses from tirzepatide-treated populations demonstrated substantial reductions in muscle fat infiltration despite decreases in muscle size, suggesting that improvements in muscle composition may accompany reductions in lean mass [94]. These findings suggest that conventional measures of lean mass alone may not fully capture the effects of GLP-1 therapy on skeletal muscle health.

Whether reductions in lean mass during GLP-1 therapy have meaningful clinical consequences remains uncertain. Most randomized trials have focused on weight loss, glycemic control, cardiovascular outcomes, and safety rather than measures of muscle function. As a result, relatively little is known about the relationship between treatment-induced changes in body composition and outcomes such as strength, mobility, exercise capacity, frailty, or independence in activities of daily living [84,95]. This distinction is important because preservation of physical function, rather than maintenance of lean mass alone, ultimately determines the clinical relevance of body composition changes.

Many discussions of lean mass loss during GLP-1 therapy assume that preserving absolute lean mass should be the primary objective. Whether this represents the most clinically meaningful endpoint remains uncertain, particularly if reductions in lean mass occur alongside improvements in adiposity, metabolic health, physical function, and muscle quality. Available evidence therefore suggests that reductions in lean mass should be considered an expected component of substantial weight loss rather than a unique consequence of GLP-1 therapy. The clinical importance of these changes, however, remains incompletely understood because data linking body composition changes to muscle strength, physical performance, and long-term functional outcomes are limited. Whether lean mass reductions observed during GLP-1 therapy represent an expected physiological adaptation to substantial weight loss or a clinically relevant threat to muscle health likely depends on patient characteristics, nutritional status, and lifestyle factors. Clarifying this distinction remains an important priority for future research and will require prospective studies incorporating comprehensive assessments of body composition, muscle strength, physical performance, dietary intake, and physical activity [83,84,94].

## 7. Sarcopenic Obesity, Frailty, and High-Risk Populations

The nutritional and functional consequences of GLP-1-based therapy are unlikely to be uniform across all individuals. Similar reductions in food intake, body weight, or lean mass may have different clinical implications depending on baseline nutritional status, muscle health, age, comorbidities, physical activity, and physiological reserve. Growing attention has focused on populations in whom nutritional inadequacies or changes in body composition may be more likely to translate into adverse clinical outcomes, particularly individuals with sarcopenic obesity, frailty, chronic disease, or pre-existing functional limitations [17,42,45].

Sarcopenic obesity is characterized by the coexistence of excess adiposity and impaired skeletal muscle health, reflected by reduced muscle mass, diminished muscle strength, impaired physical performance, or a combination of these features. Recognition of sarcopenic obesity has increased substantially in recent years because accumulating evidence indicates that the condition is associated with a greater risk of disability, cardiometabolic disease, hospitalization, and mortality than either obesity or sarcopenia alone [45,96]. Recent estimates suggest that sarcopenic obesity affects approximately 5–20% of adults, depending on the population studied and diagnostic criteria applied, with prevalence increasing substantially among older adults and individuals with chronic disease [17,45]. The recently published ESPEN and EASO consensus statement represented an important step toward standardization by proposing diagnostic criteria that integrate measures of adiposity, muscle mass, muscle strength, and physical performance [42].

The relevance of sarcopenic obesity to GLP-1 therapy extends beyond changes in body composition alone. Individuals with pre-existing deficits in muscle mass or function may have less physiological reserve to tolerate reductions in lean tissue during weight loss, particularly when accompanied by inadequate protein intake, low levels of physical activity, or other nutritional vulnerabilities [15,17,42]. In addition, obesity-related metabolic abnormalities, chronic low-grade inflammation, insulin resistance, and physical inactivity may impair anabolic responsiveness to dietary protein and exercise, thereby increasing susceptibility to functional decline [17,97]. These considerations do not imply that GLP-1 therapy causes sarcopenia; rather, they highlight the possibility that some individuals may be less resilient to nutritional or body composition changes that accompany substantial weight loss.

Ageing represents one important modifier of this risk. Progressive declines in skeletal muscle mass and strength occur throughout later life and are accompanied by anabolic resistance, a phenomenon characterized by a diminished muscle protein synthetic response to dietary protein and physical activity [98,99]. As a result, older adults generally require greater attention to nutritional adequacy and to resistance exercise during weight-loss interventions. At the same time, older adults may derive substantial benefits from intentional weight reduction, including improvements in mobility, cardiometabolic health, osteoarthritis symptoms, and quality of life [100]. The clinical challenge, therefore, lies not in avoiding effective obesity treatment but in balancing weight-loss goals with preservation of muscle health and functional capacity.

Frailty provides an additional framework for considering nutritional vulnerability during GLP-1 therapy. Although frailty has traditionally been associated with undernutrition and low body weight, obesity and frailty frequently coexist, particularly in later life [101]. Excess adiposity may contribute to reduced mobility, chronic low-grade inflammation, multimorbidity, and declining physical function, resulting in reduced physiological reserve and increased vulnerability to health stressors despite excess body weight [17,102]. In this context, nutritional inadequacies may have consequences that extend beyond body composition, influencing mobility, independence, recovery from illness, and the ability to respond to physiological stressors, all of which contribute to maintenance of functional capacity and quality of life [101,103]. Relatively few GLP-1 trials have incorporated validated assessments of frailty, physical performance, or disability as predefined outcomes, limiting current understanding of how treatment influences these clinically important domains [84,95].

An additional consideration is that obesity should not be regarded as protection against malnutrition. Individuals living with obesity frequently present with poor dietary quality, micronutrient inadequacies, reduced physical activity, and impaired muscle function despite excess energy stores, illustrating that nutritional status cannot be inferred from body weight alone [104,105,106]. Similarly, reductions in body weight do not necessarily reflect improvements in nutritional status [104,107]. Evaluation of treatment success, therefore, requires consideration of nutritional adequacy and functional outcomes alongside conventional measures of weight loss and metabolic improvement.

Current evidence does not support withholding effective obesity treatment because of concerns regarding potential muscle loss or nutritional vulnerability. Certain individuals may require closer nutritional and functional monitoring throughout treatment. This perspective shifts the clinical focus from weight reduction alone toward preservation of physiological reserve, functional independence, and long-term health. Recognition of these clinical phenotypes highlights the importance of tailoring nutritional monitoring according to individual risk rather than applying uniform follow-up strategies to all patients. The principal phenotypes associated with nutritional vulnerability during GLP-1 therapy, along with their corresponding monitoring priorities, are summarized in Table 2.

## 8. Clinical Assessment and Nutritional Management During GLP-1-Based Therapy

The increasing use of GLP-1 receptor agonists and dual GIP/GLP-1 receptor agonists has expanded the role of nutritional care in obesity management. At present, evidence remains insufficient to support a standardized nutritional monitoring pathway applicable to all individuals receiving these therapies. The available literature instead favors a risk-adapted approach, whereby the extent of nutritional assessment is determined by baseline nutritional status, treatment response, comorbidities, functional reserve, and the development of treatment-related adverse effects rather than by the pharmacological intervention itself [21,24,25,54,107,111].

Nutritional risk should be evaluated before treatment begins. A detailed dietary history may include habitual eating patterns, meal frequency, food variety, usual protein intake, alcohol consumption, previous weight-loss attempts, gastrointestinal symptoms, physical activity, and relevant comorbidities. Identifying conditions that may reduce nutritional resilience, including previous bariatric surgery, chronic gastrointestinal disease, restrictive dietary practices, food insecurity, frailty, sarcopenic obesity, or documented micronutrient deficiencies, allows follow-up to be tailored according to anticipated nutritional risk rather than applied uniformly across all patients [111,112,113].

Rather than focusing exclusively on body weight or metabolic parameters, follow-up appointments offer an opportunity to detect early changes in dietary behavior that may precede clinically relevant nutritional deterioration. Persistent nausea or vomiting, prolonged very low food intake, marked reductions in dietary variety, avoidance of protein-rich foods, fatigue, declining exercise tolerance, constipation associated with inadequate fluid or fiber intake, or new limitations in everyday activities may all indicate the need for more comprehensive nutritional evaluation. In individuals considered to be at increased functional risk, simple bedside measures, including handgrip strength, gait speed, or the five-times chair-stand test, may provide clinically meaningful information even when advanced body composition assessment is unavailable. Nevertheless, the practical aspects of incorporating functional testing into routine obesity clinics remain uncertain because access to equipment, trained personnel, and consultation time varies substantially across healthcare settings. Pragmatic screening approaches may therefore be particularly relevant in primary care and other high-volume clinical settings [42,97,107]. Brief dietary screening tools may facilitate initial evaluation of dietary adequacy, while the SARC-F provides a simple, inexpensive questionnaire for screening individuals at risk of adverse sarcopenia-related outcomes [114]. These approaches may help identify patients who warrant more detailed nutritional or functional assessment when access to comprehensive body composition or functional assessment is limited.

Current evidence also supports a selective rather than universal approach to laboratory assessment. Routine biochemical screening for micronutrient deficiencies has not been shown to benefit all individuals receiving GLP-1-based therapy and may increase healthcare costs without improving patient outcomes. Conversely, laboratory evaluation becomes increasingly relevant in the presence of persistent gastrointestinal symptoms, prolonged dietary restriction, prior bariatric surgery, chronic gastrointestinal disease, unexplained anemia, neuropathic symptoms, osteoporosis, or other clinical features suggestive of nutritional deficiency. This risk-based strategy is consistent with contemporary clinical nutrition principles while acknowledging that prospective studies evaluating optimal monitoring schedules during GLP-1 therapy remain unavailable [24,25,47,54,115].

The principal objective of nutritional management is not simply to maintain energy intake but to preserve dietary quality despite reduced appetite. In clinical practice, strategies such as emphasizing protein-rich, nutrient-dense foods, consuming smaller meals when gastrointestinal symptoms limit intake, maintaining adequate hydration, and progressively increasing dietary fiber are commonly recommended to support dietary quality during treatment. These recommendations largely reflect established principles of obesity management and clinical nutrition rather than direct evidence from GLP-1-specific intervention studies. Also, although they are biologically plausible and widely adopted in clinical practice, their effectiveness during pharmacologically induced weight loss requires prospective evaluation. Similarly, oral nutritional supplements or micronutrient supplementation appear most appropriate when adequate dietary intake cannot be achieved or when clinically relevant deficiencies have been confirmed [24,47,113].

Physical activity remains an essential component of nutritional management because preservation of muscle function depends on both adequate nutritional intake and mechanical loading. Current international recommendations advise adults to perform 150–300 min of moderate-intensity aerobic activity (or 75–150 min of vigorous-intensity activity) each week, together with muscle-strengthening activities involving all major muscle groups on at least two days per week. These recommendations remain applicable during GLP-1 therapy; however, their implementation often requires adaptation based on age, baseline fitness, mobility limitations, osteoarthritis, frailty, and patient preferences. For individuals with sarcopenic obesity or substantial functional impairment, supervised resistance training may offer greater benefit than generalized exercise advice alone, although direct evidence in GLP-1-treated populations remains limited [116,117,118].

Dietitian involvement is likely to be most valuable in patients with persistent gastrointestinal intolerance, inadequate protein intake, poor dietary variety, previous bariatric surgery, restrictive dietary practices, suspected malnutrition, or complex chronic disease. Collaboration between physicians, dietitians, physiotherapists, and exercise specialists also facilitates management of patients in whom nutritional vulnerability coexists with functional limitations. Importantly, nutritional care should be developed collaboratively with patients, recognizing that long-term adherence is influenced not only by clinical recommendations but also by individual food preferences, cultural practices, financial constraints, and personal treatment goals. Integrating these factors into shared decision-making may improve the sustainability of lifestyle modifications after pharmacotherapy initiation [111,113,119].

**Figure 3:**
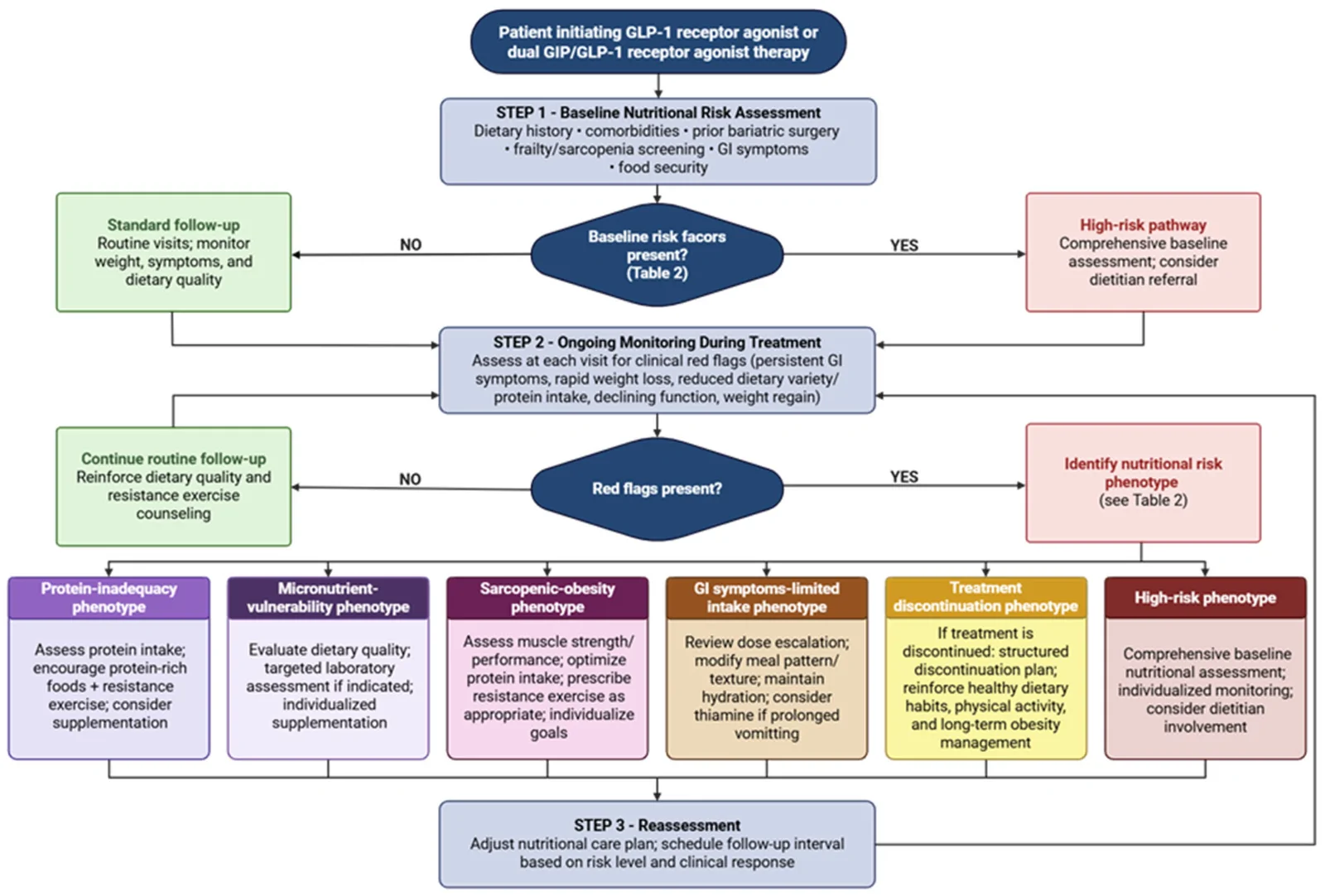
Proposed framework for nutritional risk stratification and management during GLP-1-based therapy. Patients are first assessed for baseline nutritional risk factors (Table 2); those with identified risk factors follow a high-risk pathway with comprehensive baseline assessment, while others proceed to standard follow-up. During treatment, patients are monitored at each visit for clinical red flags. When red flags are present, the corresponding nutritional risk phenotype (protein inadequacy, micronutrient vulnerability, sarcopenic obesity, GI symptom-limited intake, high-risk, or treatment discontinuation) is identified and managed according to phenotype-specific considerations. All pathways converge on periodic reassessment, with the nutritional care plan and follow-up interval adjusted according to risk level and clinical response. Created in BioRender. Milić, M. (2026), https://BioRender.com/mlanv1q (accessed on 13 August 2026).

To provide a practical overview of this risk-adapted approach, the proposed nutritional risk phenotypes and corresponding assessment, monitoring, and management considerations are summarized in Figure 3.

Even though awareness of nutritional issues during GLP-1 therapy has increased substantially, many practical questions remain unresolved. Evidence remains limited regarding the optimal frequency of nutritional follow-up, the most appropriate screening tools for detecting clinically relevant nutritional deterioration, and the cost-effectiveness of incorporating routine functional assessment or body composition analysis into obesity care. Whether structured nutritional management translates into clinically meaningful improvements in physical function, treatment persistence, and long-term health outcomes remains an important unanswered question.

## 9. Future Research Priorities

### 9.1. Defining Meaningful Nutritional Outcomes

Interpreting the nutritional effects of GLP-1-based therapies remains challenging because studies often evaluate different outcomes despite addressing similar research questions. Some studies evaluate dietary intake; others focus on body composition or biochemical markers; and relatively few incorporate measures of physical function or patient-reported outcomes. These measures describe different aspects of nutritional health and should not be considered interchangeable. Comparing results across studies is often difficult, even when similar interventions are investigated [82,84].

Methodological heterogeneity is only part of the problem. Equally important is the lack of agreement regarding which nutritional outcomes are most relevant to patients. Changes in lean mass, protein intake, or circulating micronutrients may be statistically significant without affecting daily functioning or quality of life. In contrast, relatively small nutritional changes may have important consequences in vulnerable individuals. Determining which nutritional changes are clinically meaningful remains an important challenge [82,120].

This shift is also reflected in recent international initiatives. A Delphi consensus defining a core patient-centered outcome set for obesity emphasized physical functioning, participation in everyday life, treatment burden, and health-related quality of life alongside conventional biomedical outcomes [120]. Similar conclusions emerged during the development of a core outcome set for obesity self-management interventions, where participants consistently prioritized outcomes extending beyond changes in body weight alone [121]. These initiatives were not developed specifically for GLP-1 therapy, but they provide a useful framework for future nutritional research.

Patient-reported outcomes have begun to appear in obesity pharmacotherapy trials, including the STEP program, where semaglutide improved physical functioning and weight-related quality of life [122]. Most nutritional studies continue to rely predominantly on outcomes such as body weight, body composition, or laboratory measurements, while the relationship between these markers and patient-centered outcomes remains poorly defined [16,33,38,84,123].

Collecting more nutritional variables is unlikely to resolve this problem unless greater agreement is reached regarding which outcomes are clinically meaningful, how they should be measured, and when intervention is warranted. Until then, increasingly detailed nutritional datasets are unlikely to translate into clearer clinical guidance.

### 9.2. Towards More Individualized Nutritional Research

Current evidence suggests that nutritional responses to GLP-1-based therapy are highly variable, yet this variability has received relatively little attention in clinical research. Most studies report average changes in the study population, whereas differences in baseline nutritional status, body composition, dietary quality, comorbidities, or functional reserve are examined less frequently. Although this approach is appropriate for establishing treatment efficacy, it provides only limited insight into why some individuals tolerate prolonged reductions in food intake without clinically relevant nutritional consequences, whereas others appear considerably more vulnerable [124,125].

Obesity itself is increasingly recognized as a heterogeneous chronic disease rather than a single clinical entity. Similar body weight or body mass index may reflect markedly different metabolic profiles, eating behaviors, body composition, and nutritional status. This heterogeneity is likely to influence nutritional responses during GLP-1 therapy, yet it has rarely been incorporated into study design or participant stratification [124,126].

Future research would therefore benefit from moving beyond conventional demographic subgroup analyses towards more comprehensive nutritional phenotyping. Baseline dietary quality, muscle health, gastrointestinal symptoms, previous bariatric surgery, multimorbidity, and socioeconomic circumstances are all plausible modifiers of nutritional risk, but they are rarely considered collectively. Integrating these factors into prospective studies may improve the identification of individuals most likely to benefit from tailored nutritional support rather than uniform follow-up strategies [124,127].

Individualization should also extend beyond biological characteristics. Access to dietetic care, affordability of nutrient-dense foods, health literacy, and opportunities for physical activity differ substantially across populations and healthcare systems. These factors are likely to influence nutritional outcomes during treatment but are largely absent from current research on obesity pharmacotherapy. Better characterization of these contextual determinants would improve both the interpretation of clinical trials and the generalizability of their findings [124].

Rather than asking whether GLP-1 receptor agonists influence nutritional status, future investigations may achieve greater clinical relevance by identifying which patients are most likely to develop nutritional compromise, which factors modify this risk, and which interventions are effective within specific clinical contexts. Such an approach would move nutritional research closer to the principles of precision medicine while acknowledging that individualized nutritional care cannot be based on pharmacological treatment alone.

### 9.3. From Surrogate Outcomes to Patient-Centered Research

Future nutritional research should move beyond describing changes in dietary intake, body composition, or biochemical markers and determine how these changes influence outcomes that matter to patients. Although surrogate markers remain important, they provide only limited information regarding treatment burden, physical function, symptom severity, or the ability to maintain healthy eating behaviors over time. Greater integration of patient-reported outcomes into nutritional studies may therefore provide a more comprehensive assessment of treatment effectiveness [122,123].

Another area requiring further attention is nutritional monitoring outside controlled clinical trials. Digital dietary assessment, image-assisted food records, and other technology-assisted approaches may facilitate repeated assessment of dietary intake while reducing participant burden. These tools have shown encouraging results in nutritional research, but their role during GLP-1 therapy has not yet been established [128,129].

The next challenge is not simply to generate more nutritional data, but to determine which findings meaningfully inform clinical decision-making and improve patient care. Only then can nutritional evidence be translated into practical and sustainable models of nutritional management during GLP-1-based therapy.

### 9.4. Interventional Trials of Combined Protein Optimization and Resistance Exercise

A major evidence gap is the lack of completed randomized controlled trials specifically evaluating combined protein optimization and structured resistance exercise during GLP-1-based therapy. Although exercise interventions have been investigated alongside GLP-1 receptor agonist treatment [130], current recommendations to combine adequate protein intake with resistance exercise for preservation of muscle mass are supported largely by evidence from general weight-loss and aging populations and by expert guidance [24,131]. It therefore remains uncertain whether this combined approach attenuates lean mass loss or improves muscle strength, physical performance, and patient-centered outcomes specifically in individuals receiving GLP-1-based therapy. Importantly, this evidence gap is beginning to be addressed: the ongoing LEAN-PREP randomized controlled trial is evaluating resistance exercise, increased protein intake, and their combination in adults with obesity initiating semaglutide or tirzepatide therapy [132]. Until results from this and similar trials become available, adequately powered randomized controlled trials with predefined body composition, functional, and patient-centered outcomes remain necessary to establish the effectiveness of this approach and inform more specific recommendations regarding protein intake and exercise prescription.

## 10. Conclusions

The expanding use of GLP-1-based therapies has brought nutritional considerations into focus as an integral component of obesity management. Although these medications achieve substantial and clinically meaningful weight loss, reductions in energy intake may also influence dietary quality, protein intake, micronutrient adequacy, body composition, and muscle health. Current evidence indicates that these changes are not uniform across individuals and are shaped by baseline nutritional status, clinical characteristics, and treatment tolerance.

Body weight alone provides only a limited picture of nutritional health during treatment. A more comprehensive assessment that considers dietary intake, body composition, muscle function, physical performance, and individual clinical context is likely to provide greater clinical value, particularly in patients with pre-existing nutritional vulnerability. At the same time, available evidence does not support routine intensive nutritional monitoring or empirical supplementation for all individuals receiving GLP-1-based therapy. Instead, nutritional care should remain individualized and proportionate to clinical risk. Assessment of baseline nutritional status and dietary quality may help identify pre-existing deficiencies and other nutritional vulnerabilities before treatment initiation. When clinically indicated, these abnormalities should be addressed before or concurrently with GLP-1-based therapy rather than assumed to have arisen as a consequence of treatment.

Several important questions remain unresolved. Evidence linking nutritional changes with functional outcomes, disability, treatment persistence, and long-term health remains limited, and standardized approaches to nutritional assessment have yet to be established. Future research should focus not only on describing nutritional changes but also on identifying clinically meaningful outcomes, determining which patients benefit most from targeted nutritional support, and evaluating practical models of nutritional care for routine clinical practice.

As GLP-1-based therapies continue to reshape obesity treatment, nutritional management should evolve alongside pharmacotherapy. Integrating nutritional assessment into comprehensive obesity care can support both the safety and the long-term effectiveness of treatment, while acknowledging that nutritional needs vary among individuals.

## Figures and Tables

**Table 1:** Micronutrients of clinical interest during GLP-1-based therapy: evidence summary, potential clinical relevance, high-risk groups, and monitoring considerations.

Micronutrient	Evidence Summary and Study Context	Potential Clinical Relevance	Groups at Increased Risk	Monitoring/Management Considerations	References
Vitamin D	Retrospective claims cohort (*n* = 461,382): incident coded vitamin D deficiency was documented in 7.5% within 6 months and 13.6% within 12 months after GLP-1RA initiation. Patients with previously coded nutritional deficiencies were excluded, but baseline biochemical 25(OH)D status was unavailable.	Bone health, calcium homeostasis, muscle function, immune regulation	Older adults, individuals with obesity, limited sun exposure, low dietary intake, postmenopausal women	Consider baseline 25(OH)D in at-risk individuals; repeat testing according to risk and clinical context; supplement according to deficiency guidelines	[52,53,55,56,57,58]
Iron	Register-based study in individuals with T2D and hemochromatosis: GLP-1RA exposure was associated with lower ferritin levels than SGLT2 inhibitor exposure. Separately, a cross-sectional study of 69 GLP-1RA users reported mean iron intake below DRI values (12.1 mg/day); pre-treatment dietary intake was not assessed.	Iron deficiency, anemia, fatigue, reduced physical performance	Premenopausal women, individuals with heavy menstrual bleeding, vegetarians/vegans, those with low dietary iron intake or pre-existing low iron stores	CBC, ferritin, and transferrin saturation when clinically indicated; supplementation according to confirmed deficiency and clinical context	[33,38,47,56,59,60]
Calcium	Cross-sectional dietary assessment of 69 GLP-1RA users: mean calcium intake was below DRI values (863 mg/day); pre-treatment dietary intake was not assessed.	Bone health, muscle contraction, fracture risk in vulnerable groups	Older adults, postmenopausal women, individuals with low dietary calcium intake, lactose intolerance, or vitamin D deficiency	Assess dietary calcium intake; serum calcium has limited value for assessing dietary adequacy; consider bone-health assessment in high-risk patients	[33,47,55,56,61,62]
Vitamin B12	GLP-1-specific prevalence of biochemically confirmed vitamin B12 deficiency has not been established, and available evidence does not clearly distinguish pre-existing from treatment-emergent deficiency.	Megaloblastic anemia, neuropathy, fatigue, cognitive symptoms	Older adults, vegans/vegetarians, metformin users, individuals with a history of bariatric surgery	Serum vitamin B12, with methylmalonic acid and/or homocysteine when clinically indicated; assess concomitant metformin use and other risk factors; supplement when deficiency is confirmed or strongly suspected	[47,56,63,64]
Thiamine	Case reports and pharmacovigilance data have described thiamine deficiency and Wernicke encephalopathy during GLP-1-based therapy, most often in the context of persistent gastrointestinal symptoms, markedly reduced intake, or rapid weight loss; population incidence is unknown.	Wernicke encephalopathy, neuropathy, cardiovascular manifestations in severe deficiency	Persistent vomiting, markedly reduced intake, rapid weight loss, alcohol use disorder, history of bariatric surgery	Routine screening is not indicated for all GLP-1 users; promptly assess and treat suspected thiamine deficiency in individuals with persistent vomiting, markedly reduced intake, rapid weight loss, or neurological symptoms.	[47,65,66,67,68]
Folate	GLP-1-specific longitudinal data on folate status are limited, and the incidence of treatment-emergent folate deficiency has not been established.	Megaloblastic anemia, elevated homocysteine, pregnancy-related risks	Women of reproductive age, individuals with restrictive diets or low intake of folate-rich foods, and those with pre-existing malabsorption or other established risk factors	Assess dietary folate intake; serum and/or RBC folate when clinically indicated; supplementation according to standard recommendations and individual clinical context	[47,55,56,69]
Magnesium	Cross-sectional dietary assessment of 69 GLP-1RA users: mean magnesium intake was below DRI values (266 mg/day); pre-treatment dietary intake was not assessed. GLP-1-specific prevalence of biochemical magnesium deficiency remains unknown.	Neuromuscular function, glucose metabolism, cardiovascular health; deficiency may contribute to muscle cramps and fatigue	Older adults, individuals with T2D, users of diuretics or proton pump inhibitors, and those with low dietary magnesium intake or limited dietary variety	Assess dietary magnesium intake and relevant medications; serum magnesium may not fully reflect total body stores; laboratory evaluation and supplementation when clinically indicated	[33,38,47,56,70]
Zinc	Cross-sectional dietary data did not indicate inadequate mean zinc intake relative to DRI values; GLP-1-specific longitudinal data on biochemical zinc status and treatment-emergent deficiency are lacking.	Immune function, wound healing, taste changes, appetite regulation	Vegans/vegetarians, individuals with low protein or low dietary zinc intake, malabsorptive disorders, and other conditions associated with impaired zinc status	Assess dietary zinc intake and clinical risk factors; laboratory evaluation when deficiency is suspected; avoid prolonged high-dose zinc supplementation because of the risk of copper deficiency	[33,47,55,71,72]

Note: Evidence summaries distinguish dietary inadequacy, diagnostic coding, biochemical measurements, and study design where these data were available. Cross-sectional findings should not be interpreted as treatment-emergent deficiencies in the absence of pre-treatment assessment. Abbreviations: 25(OH)D, 25-hydroxyvitamin D; CBC, complete blood count; GLP-1, glucagon-like peptide-1; GLP-1RA, glucagon-like peptide-1 receptor agonist; DRI, dietary reference intake; RBC, red blood cell; SGLT2, sodium-glucose cotransporter-2; T2D, type 2 diabetes.

**Table 2:** Clinical phenotypes associated with nutritional vulnerability during GLP-1-based therapy.

Clinical Phenotype	Typical Clinical Presentation	Clinical Clues	Primary Clinical Priority	Suggested Clinical Approach	References
Protein-inadequacy phenotype	Marked appetite suppression with reduced protein intake during weight loss	Rapid weight loss, reduced appetite, declining muscle strength, low dietary protein intake	Preserve skeletal muscle and functional capacity	Assess dietary protein intake; encourage protein-rich foods and resistance exercise; consider protein supplementation when dietary intake remains inadequate	[15,24,33,38]
Micronutrient-vulnerability phenotype	Reduced dietary variety and low nutrient density resulting from persistent energy restriction	Monotonous diet, prolonged gastrointestinal symptoms, restrictive eating pattern, pre-existing nutritional deficiencies	Maintain nutritional adequacy and prevent clinically relevant deficiencies	Evaluate dietary quality; perform targeted laboratory assessment when clinically indicated; provide individualized supplementation only when appropriate	[16,47,52,104]
Sarcopenic-obesity phenotype	Excess adiposity accompanied by impaired muscle strength and/or physical performance	Difficulty rising from a chair, slow gait, reduced grip strength, physical inactivity	Preserve muscle function and physical independence	Assess muscle strength and physical performance; optimize protein intake; prescribe resistance exercise; individualize weight-loss goals	[42,54,84,94]
GI symptom-limited intake phenotype	Persistent gastrointestinal symptoms limiting food intake	Persistent nausea, vomiting, early satiety, food aversion, dehydration	Restore adequate nutritional intake while minimizing symptoms	Review dose escalation; modify meal pattern and food texture; maintain hydration; consider thiamine replacement in patients with prolonged vomiting	[47,54,66]
High-risk phenotype	Pre-existing nutritional vulnerability before initiation of GLP-1 therapy	Older age, frailty, previous bariatric surgery, chronic gastrointestinal disease, chronic kidney or liver disease, food insecurity	Early recognition of patients requiring individualized nutritional care	Perform comprehensive baseline nutritional assessment; involve a registered dietitian when appropriate; individualize monitoring and follow-up	[11,42,54,107]
Treatment discontinuation phenotype	Weight regain following treatment discontinuation	Rapid weight regain, deterioration in dietary habits, reduced physical activity	Maintain long-term metabolic and functional benefits	Develop a structured discontinuation plan; reinforce healthy dietary habits, physical activity, and long-term obesity management	[108,109,110]

Footnote: These phenotypes are conceptual clinical categories intended to facilitate nutritional risk assessment during GLP-1-based therapy. They do not represent formal diagnostic entities, and individual patients may exhibit features of more than one phenotype. Clinical assessment should be individualized according to baseline nutritional status, comorbidities, treatment response, and functional capacity. Abbreviations: GI, gastrointestinal; GLP-1, glucagon-like peptide-1.

## Data Availability

No new data were created or analyzed in this study. Data sharing is not applicable to this article.

## Abbreviations

The following abbreviations are used in this manuscript:

GLP-1	glucagon-like peptide-1
T2D	type 2 diabetes
MeSH	Medical Subject Headings
GIP/GLP-1	glucose-dependent insulinotropic polypeptide/glucagon-like peptide-1
DXA	dual-energy X-ray absorptiometry
